# Insect sting allergy. A study from 1980 to 2003 of patients who started treatment with venom immunotherapy between 1980 and 1998

**DOI:** 10.1186/1476-7961-3-12

**Published:** 2005-08-19

**Authors:** Rolf Haye, Liv Kari Døsen

**Affiliations:** 1 Department of otolaryngology, Rikshospitalet-Radiumhospitalet HF University of Oslo 0027 Oslo, Norway

## Abstract

**Background:**

Previously we treated patients with insect sting allergy with venom immunotherapy (IT) using whole body insect extracts. From 1980 we changed to insect venoms. The purpose of this study was to analyse data from the patients in order to improve our treatment.

**Methods:**

This is an open, single centre study on patients treated with venom IT 14 years or older with a history of a systemic allergic reaction to an insect sting, a positive skin prick test (SPT) or a positive RAST and willingness to comply with five years of IT. Clinical and laboratory data were registered prospectively at the start of IT and after five years of treatment until 2003 on patients who started IT between 1980 and 1998. Questionnaires were answered in 1989, 1993 and 2003. Statistical analysis was done with Pearson's chi square, Fisher's exact or the t-test.

**Results:**

Of 315 patients treated, 44 were given bee, 248 common wasp and 23 both venoms. Of the common wasp sting incidents 5.5 % resulted in a severe allergic reaction (SAR) during adequate IT and 22% after cessation. Seventy-one per cent of the patients carried epinephrine. Precautionary steps were taken by 77% of the patients during or after inadequate IT. On or after adequate IT 83% felt completely or substantially safe. Surprisingly 29 % of those inadequately treated felt safer and 50% were satisfied with having had the opportunity to be treated. The SPT became negative in 68% of the wasp allergic patients after five years of adequate IT. Increased risk of experiencing SAR to a future sting in wasp allergic patients after cessation of adequate IT was significantly associated with a SAR due to IT during the rush regimen. SAR due to IT occurred very rarely during maintenance dosing.

**Conclusion:**

Adequate venom IT is very effective while ongoing but somewhat less effective after cessation, while inadequate treatment gives poor results. More of our patients should complete five years of IT and some should continue IT. The type of reaction to IT during incremental dosing may be of help in deciding who should continue beyond five years. Maintenance IT may be taken over by the general physician.

## Background

After the study of Hunt et al [1] was published in 1978 showing the ineffectiveness of whole body insect extracts, we stopped using whole body insect extracts in the treatment of patients with insect sting (hymenoptera) allergy. When venom from bee and common wasp (vespula sp) became available in Norway in 1979, we started skin testing patients allergic to insect stings and thereafter treating those who had a positive skin prick test (SPT) or positive specific IgE reaction (RAST) with immunotherapy (IT) using insect venoms.

The purpose of this study was to examine the short and long term effectiveness; side effects; causes for cessation of IT; the serological data and the SPT results and quality of life of our insect sting allergic patients, in order to help us improve our treatment. The patients started IT between 1980 and 1998. The results from the first eight years of treatment were published in 1988 [2], and are incorporated in the present study.

## Materials and methods

In Norway there are about 20 different species of wasps, of which vespula vulgaris and germanica (common wasps) are frequently seen. Vespa crabro has not been observed in Norway [3]. From a previous study we found that we could use venom from common wasp (vespula sp) as the sole wasp venom to our wasp allergic patients [3]. In 1979 we established the concentration that discriminates insect sting allergic patients from normal individuals and found this to be 100 microgram pr. ml for the SPT and 1 microgram pr ml for the intracutaneous test (IC) [4] the latter corresponding to data from the study of Hunt et al [5]. We found the sensitivity to be almost equal for both tests [4] and as SPT is the preferred method for skin testing in Norway, we chose SPT as the standard method

Patients who had a history of an immediate systemic allergic reaction, with vascular and/or respiratory symptoms, angioedema of the head and neck or symptoms from two organ systems, to a sting from bee and/or wasp and who were willing to comply with a minimum of five years of treatment were offered IT. The SPT or RAST reaction had to be positive to the respective venom. For SPT we used Pharmalgen (ALK) venoms at 100 microgram pr. ml concentration and Soluprick (ALK) for inhalant allergens. Histamine chloride served as reference, 1 mg/ml for insect venoms during the whole study. For the first five years of the study the commercially available SPT test for inhalant allergens used 1 mg/ml histamine as reference and later 10 mg/ml. The concentration of the inhalant allergens was adjusted accordingly. If the size of the wheal of the SPT area was 50% or larger than that caused by histamine and had a minimum diameter of 3 mm larger than the control, it was recorded as positive. Serum analyses of total and specific IgE were done at a laboratory using Pharmacia RAST from 1980 to1987, CAP from1988 to1998 and DCP Alastat from 1999 to 2003. In addition analyses of haemoglobin, leukocytes, CRP, creatinine, ASAT, ALAT and serum electrophoresis were undertaken. Serum tryptase was not analysed, however none of the patients had urticaria pigmentosa. The IT was performed with Pharmalgen (ALK) venoms. The injections were given subcutaneously on the lateral part of the upper arm. Aspirations were done at the beginning and during the injection. We used a rush regimen of five days duration giving a starting dose of 0.1 microgram venom, doubling this every 2 hours for three days. The dose was increased more slowly on day four and five aiming for 50 micrograms or more for the last injection. Thereafter the venom was given at weekly intervals gradually increasing the dose to 0.1 mg and the interval to six weeks. Seven patients received Alutard (ALK) venoms. Many patients were referred back to their general practitioner to continue the IT after having reached 0.1 mg as their maintenance dose. A few patients who only tolerated 50 micrograms venom used this as their maintenance dose even if this is less effective. If they did not tolerate 50 micrograms of venom after up to three years of IT, the treatment was ended. Patients who tested positive to both bee and common wasp venoms were treated with both venoms. Beekeepers and their relatives who were allergic to bee stings were not given IT if they abandoned bee keeping and there was no bee keeping within 10 km. Patients were instructed to keep epinephrine at hand and for those who had not reached the dose of 0.1 mg venom, to inject it immediately after a sting. Having reached this dose they should only inject if symptoms occurred.

Five years after the start of IT the patients were asked to return for clinical examination, SPT and serological tests, regardless of status of completion of IT. Many patients also met for an extra consultation at a later date for the same procedures. Information was also obtained at the time of renewal for prescription of venom. Questionnaires asking for the patients reactions to stings, and reaction to and length of IT were answered by mail and/or by telephone interview in 1989, 1993 and 2003. The 2003 questionnaire also included questions on precautionary steps and safety feeling. We divided our patients into two groups according to the duration and dosage of the IT. IT is considered adequate if the maintenance dose is 0.1 mg venom and at cessation has lasted a minimum of five years. It is inadequate when the dose is less than 0.1 mg or at cessation of a shorter duration than five years. SAR (serious allergic reaction) is defined as a respiratory and/or vascular reaction with an additional feeling of impending disaster or other serious symptom or requiring treatment with epinephrine.

Statistical analysis was done with Pearson's chi square or Fisher's exact test. For the duration of IT two samples t-test was used.

## Results

Three hundred and fifteen patients were included in the study, 151 males (48%) and 164 females (52%). The age varied from 14 to 73 with a mean of 41.1 years. Forty four patients (14%) were given bee, 248 (79%) common wasp (vespula sp.) and 23 (7%) both venoms. Table 1 shows the most serious symptom the patients had experienced from a sting.

According to the classification of Mueller, 306 were classified as having a grade three or four and nine a grade two or lower reaction. We have grouped together grades three and four as we in many cases found it difficult to adequately classify the patients from the case histories. In stead we tried to differentiate whether the reaction was vascular, respiratory or combined vascular-respiratory (Table 1). The SPT for inhalant allergy was positive in 32.5 % of the patients, but only 21% reported having a respiratory allergy.

**Table 1:** Main Symptom

Insect\Symptom	Vascular and respiratory	Vascular	Respiratory	Angioedema head/neck	Skin + gastrointest.	Total
Bee	12	18	13	1		44
Common wasp	87	110	46	3	2	248
Bee/common wasp	6	8	6	3		23
Total	105	136	65	7	2	315

Up to 2003 twenty one patients have died of causes unrelated to insect allergy. Information regarding these patients obtained prior to their deaths is included in the results. At the five years recall 92.2% met for consultation and an additional 43.9% at a later date. The response rate to the questionnaires was 98.3% in 1989, 90.7% in 1993 and 88.6% in 2003.

Several patients have experienced a reaction to the venom injections. Their most serious reaction during incremental dosing and during maintenance is recorded in Table 2. Nearly half of the patients had a reaction and 23.5% a systemic allergic one. SAR occurred more than once in a few patients although we adjusted further dosing carefully. SAR occurred in 45 patients 14.2% (12 males, 33 females) (33 allergic to wasp, and 12 to bee) during increasing dosing.

**Table 2:** Number of patients reacting to immunotherapy

Type of reaction	During incremental dosing	During maintenance dosing
Large local reaction	14	5
Itching in throat or nose / vomiting	18	1
Urticaria	11	2
Sedation	51	25
SAR	45	5
Joint / muscle pain	21	26
Tachycardia	10	1
Total	170	65

Several patients experienced joint or muscle pain. In five cases swelling of joints was related to the injections disappearing later but sometimes steroids were given to alleviate the symptoms. In other cases rheumatoid arthritis or osteoarthritis proved to be the cause of these symptoms. During maintenance dosing SAR in one bee allergic patient occurred when an attempt was made to increase the dose above 0.1 mg, in one wasp allergic patient due to a higher than planned dose after a prolonged interval of IT, in one patient who received both venoms at the second maintenance dose, in one wasp allergic patient after a few maintenance doses and in one case there was an unexplained syncope one day later. In three of these cases there was a change in allergic sensitivity so that it was impossible later to reach an adequate maintenance dose again and IT was abandoned.

Seventy-seven patients (24%) did not complete the minimum of five years of IT. The reasons for stopping treatment before five years are given in Table 3. The most serious reaction causing cessation occurred in a female patient who had a cardiac arrest without prior warning at day four, 20 minutes after receiving 30 microgram common wasp venom as the second dose that day. It was difficult to resuscitate her, but she has recovered completely. Another patient received the dose accidentally in an intravenous drip. In the group other causes for stopping IT one was a patient afraid of acquiring AIDS from the venom injections and three patients afraid of receiving injections during pregnancy although we recommended continuation.

**Table 3:** Causes for cessation of immunotherapy before 5 years. Number of patients

Problems due to work or change of domicile	15
Reactions to immunotherapy	36
Other serious illness	7
Death of other causes	1
Own initiative	11
Tolerated multiple stings	1
Others	6
Total	77

Eighteen patients are still treated.

Seventy one per cent of the patients keep epinephrine at hand. No difference was seen whether or not the patients had completed five years of treatment. Of those inadequately treated 76 % take precautionary steps to avoid being stung, whereas only 57.4% of those adequately treated do so. Table 4 lists the different precautionary steps taken.

**Table 4:** Precautionary steps taken. Number of patients

	Total
Never barefoot	126
No perfume	84
Never drinking/eating outdoors	73
Never hiking alone	46
Always window screen	17
Mobile phone present	6
Others	16
Unknown	47

In Table 5 the feeling of safety as experienced by the patients according to the status of their IT is shown. Eighty-three per cent of the adequately treated patients feel completely or substantially safe and surprisingly 29% of the inadequately treated. Those who feel unsafe although having completed adequate IT had either experienced a SAR to a sting during treatment or were discouraged by having a positive SPT reaction at the fifth year, even if informed that this is not uncommon. Ninety-four per cent of the adequately treated patients found the investment in time, expenses and effort worthwhile (Table 6). Those who did not benefit from the IT regarded the inconvenience of IT as bothersome.

**Table 5:** Feeling of safety. Number of patients

	Adequate treatment	Inadequate treatment	Total
Complete	54	4	58
Substantially	118	14	132
Somewhat	21	19	40
None	14	24	38
Unknown	28	19	47
Total	235	80	315

**Table 6:** Worth the effort. Number of patients

	Adequate treatment	Inadequate treatment	Total
Yes	192	31	223
No	11	29	40
Unknown	32	20	52
Total	235	80	315

Reactions to field stings during treatment in patients allergic to common wasp as reported by the patients are presented in Table 7. The number of stings per incident was low, usually only one and never more than four. We have recorded the reactions to each incident regardless of the number of stings. SAR occurred in 55% of the incidents when the venom dose was below 0.1 mg. At full maintenance dosing (0.1 mg venom) only 5.5% of the incidents resulted in a SAR. Epinephrine was self administered in most cases of SAR and also in some cases where no reaction occurred (table 7). A few times the reaction occurred so rapidly that the patients did not have time enough to administer it. In bee allergic patients six of 13 patients had a SAR while inadequately treated, but only one of 14 in adequately treated ones. In addition two beekeepers had multiple stings without reaction.

**Table 7:** Reaction to sting in common wasp allergic patients during IT. Number of times stung

Type of reaction	Inadequate dosage	Adequate dosage
No / local swelling	7 (1)	132 (23)
Sedation	2 (1)	25 (10)
Itching / urticaria etc.	0	7
SAR	14 (14)	9 (7)
Joint / muscle pain	0	3 (3)
Tachycardia	0	2 (1)
Total	23 (16)	178 (44)

In parenthesis number of times adrenaline was administered.

We have also registered the reactions to stings after cessation of IT. In inadequately treated patients SAR occurred in 19 of 45 (42%) of the sting incidents in wasp allergic patients and in four of 19 (21%) in bee allergic ones. After cessation of adequate IT in common wasp allergic patients (Table 8) SAR occurred in 22% of all the sting incidents. The reaction rate was higher after five years of cessation. None of the bee allergic patients experienced a SAR after cessation of IT.

**Table 8:** Reaction to sting after adequate immunotherapy of 5 or more years' duration in common wasp. Number of times stung

Type of reaction	0–5 years after cessation	More than 5 years after cessation	Total
No / local swelling	61 (3)	14	75 (3)
Itching / urticaria etc.	5 (2)	0	5 (2)
Sedation	7 (3)	1	8 (3)
SAR	19 (11)	7 (5)	26 (16)
Joint pain	0	0	0
Tachycardia	4 (1)	0	4 (1)
Total	96 (20)	22 (5)	118 (25)

In parenthesis: Number of times adrenaline was administered.

The SPT became negative in 68% of the patients allergic to common wasp having completed adequate IT, and only in 42% of inadequately treated ones (Table 9). For bee allergic patients the SPT became negative in 74.3% of the adequately treated ones.

**Table 9:** SPT at start and after 5 years in bee and wasp allergic patients

Bee-allergic	SPT grading		
Year	Neg.	Pos.	Total

0	0+*0*	40+*23*	40+*23 *= 63
5	29+*11*	10+*11*	39+*22 *= 61

Wasp-allergic			
Year	Neg.	Pos.	Total

0	1	208+*43*	209+*43 *= 252
5	142+*18*	67+*25*	209+*43*= 252

Italics: Number of patients incompletely treated.

Total IgE did not change appreciably during the five years, see Table 10.

**Table 10:** Total IgE. Patients allergic to common wasp

kU/ml	< 122	123 – 300	> 300	Total
Year: 0	132 + *26*	29 + *8*	15 + *7*	176 + *41 *= 217
Year: 5	131 + *27*	24 + *8*	21 + *5*	176 + *40 *= 216

In italics: Number of patients incompletely treated.

No changes that could be attributed to the IT were seen in haemoglobin, creatinine, ASAT, ALAT, CRP or serum electrophoresis.

Table 11 lists a comparison between those who did not experience any reaction to a sting after cessation of adequate IT (group A) and those who experienced a SAR (group B) in patients allergic to common wasp. A statistical analysis was made on the following data, age, gender, symptom at inclusion, SPT to inhalant allergens, SPT, RAST and total IgE at five years of treatment, reaction due to incremental IT and duration of IT, to assess the prognostic value for a SAR to future stings after cessation of IT. Only the reaction to IT during incremental dosing had a prognostic value in assessing the reaction to a future sting.

**Table 11:** Comparison between 2 different groups of patients with allergy to common wasp according to reaction to sting after cessation of it

		A N = 50		B N = 17		p values
			
		No.	%	No	%	
Age	≤ 29	10	20	1	6	0.434
	30–59	34	68	14	82	
	≥ 60	6	12	2	12	
Gender	Male	33	66	9	53	0.391
	Female	17	34	8	47	
Inclusion symptom	Vascular	20	40	6	35	0.731
	Respiratory	13	26	3	17	0.485
Inclusion symptom	Vascular + respiratory	16	32	8	47	0.38
SPT pos. inhalation allergens		15	30	6	35	0.684
Reaction to rush regimen	No / local swelling	29	58	7	41	0.020
	SAR	4	8	6	35	0.013
Pos. SPT to venom 5 th year		16	32	9	53	0.123
Venom RAST, pos. 5 th year		26/48	54	11/14	79	0.129
Total IgE ≥ 123, 5 th year		9/44	20	6/14	43	0.158
Duration of IT in years		7.1 (5–15)		6.5 (5–10)		0.156

A: Patients allergic to common wasp who have had a minimum of 5 years of immunotherapy at full dose and have not had any reaction to subsequent stings.

B: Patients allergic to common wasp, who have had at least 5 years of immunotherapy at full dose, but after cessation have had serious reactions to stings.

## Discussion

This study was started in 1980 and was ongoing for many years. However the main author has been in charge during the whole period. The clinical information and laboratory data have been continuously registered. Some of the test procedures have changed. At the start the SPT kits contained histamine 1 mg/ml as reference and inhalant allergens corresponding to that (1 HEP). In 1985 histamine 10 mg/ml and allergens corresponding to this (10 HEP) became standard. As we have graded the reaction according to the corresponding reference, we believe that the results are comparable for inhalant allergens. As we chose to use 0.1 mg venom as basis for the SPT, we continued to use histamine 1 mg/ml as reference and we have therefore been able to compare the test results throughout the years.

The RAST results however may not be directly comparable as the laboratory changed their methods. The CAP system is more sensitive than the original RAST [6] so that the conversion factor from this study was used. The DPC AlaStat method has in unpublished data in-house shown to be comparably sensitive to CAP.

It is estimated that in the Nordic countries a patient in average gets a sting every tenth year [4,6]. That means that the untreated patient would expect SAR to several future stings.

This study has shown that ongoing IT for common wasp is very efficient when the maintenance dose is 0.1 mg, as also seen in other studies [1,8] and for bee even better than others [9]. The results may have been slightly different as some patients who did not experience any reaction to a sting still injected epinephrine.

We strive to get as high a percentage of patients to complete five years of IT as seen in other studies [10] because the results of our inadequately treated patients are poor and do not seem to differ from untreated ones [11]. After cessation of IT the results for inadequately treated patients are unfavourable compared to adequately treated ones.

However, the adequately treated wasp allergic patients experienced a SAR to sting after cessation of IT more frequently than has been found in other studies [12]. We have no explanation for this. Some of the patients should therefore preferable continue IT indefinitely. Such selection is difficult. According to other studies the following symptoms and signs have been found to be risk factors for experiencing SAR at a future sting after cessation of adequate IT [13]: severity of pre-treatment reaction, safety and efficacy of IT, duration of IT, and high sensitivity with the IC test. Specific IgE has in some but not all studies [13,14] been found predictive. We, however, have only found SAR due to IT during incremental dosing to be significantly predictive. Using this criterion we may be able to offer continued IT to some patients and still keep the total number treated low. The rest of the patients will have to rely on epinephrine.

Epinephrine offered as the only treatment instead of IT seems to reduce quality of life [15]. This has also been found when recommended to patients given IT [16]. We believe that carrying epinephrine is a sound safety precaution as our data show that the patients were able to use it when needed. We are satisfied that a majority of our patients carry epinephrine and feel completely or substantially safe even carrying epinephrine. This has fortunately also been seen in other studies [17,18].

We have encountered several side effects during IT. The percentage of patients with a systemic reaction during incremental dosing is comparable to the EAACI multicenter study [19] although we did not pretreat our patients with antihistamines. The percentage of patients experiencing a SAR during incremental dosing is higher. One reason for this may due to the higher percentage of females in this study as the reaction rate to IT in females was higher than in males in this and the other study [19]. Our rush regime leads to a high accumulated dose on the fourth or fifth day. This was in another study [20] found to be a cause for serious reactions. We therefore planned to modify our IT schedule on day four and five to reduce the number of reactions. However, venoms are presently only available in depot extracts in Norway. The very few reactions during normal adequate venom maintenance dosing allows us to continue our practice of having the patients' local physicians continue the IT if they take the necessary precautions.

Besides the allergic reactions we found sedation (fatigue) and particularly joint and muscle pain to be an obstacle to further IT. Headache and fatigue is well known [21] but did not lead to cessation of IT in our study, although some patients had to abstain from work on the day of injection and sometimes also the next day. Little attention has been paid to joint/muscle pain which in this study led to cessation of IT in some cases. Although we suspect this to be an immune disease, we were unable to confirm it.

A large majority of the patients felt that the investment in time, effort and expenses was worthwhile if they completed IT. This is comparable to reports by Oude Elbrink et al. [18] and Røsjø et al. [17]. Surprisingly 50% of those that did not complete adequate IT also felt so. We believe that these patients were grateful for having had the opportunity to be treated even though it was not adequate.

## Conclusion

Ongoing venom IT is very effective when adequately performed and the majority of patients feel substantially safer. Most of them found the effort very worthwhile. We should endeavour to get more of the patients to complete five years of IT as the results both while ongoing and after IT is much better for the adequately than for the inadequately treated patients. Still after cessation of adequate IT 22% of the wasp sting incidents resulted in a SAR. Fortunately the majority of patients keep epinephrine at hand and were able to use it when needed. Maintenance IT of 0.1 mg of single venom may be taken over by the general physician. Only the following parameter, the reaction to IT during incremental dosing was statistically significant for predicting the reaction to a future sting after cessation of adequate IT. This may help in deciding whether a patient should continue IT after five years or not.

## Competing interests

The author(s) declare that they have no competing interests.

## Authors' contributions

RH performed the consultations, registrations and follow ups of patients from 1980 to 2003, drafted and took care of the questionnaires from 1989 and 1993, compiled the data, drafted the tables and is the main author. LKD participated in the consultations and the follow up from 2001–2003, designed and took care of the 2003 questionnaire, participated in compiling the data and in drafting and revising the manuscript.
